# Pre-Processing of Point-Data from Contact and Optical 3D Digitization Sensors

**DOI:** 10.3390/s120101100

**Published:** 2012-01-20

**Authors:** Igor Budak, Djordje Vukelić, Drago Bračun, Janko Hodolič, Mirko Soković

**Affiliations:** 1 Faculty of Technical Sciences, University of Novi Sad, Trg Dositeja Obradovica 6, 21000 Novi Sad, Serbia; E-Mails: vukelic@uns.ac.rs (D.V.); hodolic@uns.ac.rs (J.H.); 2 Faculty of Mechanical Engineering, University of Ljubljana, Askerceva 6, 1000 Ljubljana, Slovenia; E-Mails: drago.bracun@fs.uni-lj.si (D.B.); mirko.sokovic@fs.uni-lj.si (M.S.)

**Keywords:** reverse engineering, 3D digitization, point data pre-processing, contact sensor, optical triangulation sensor

## Abstract

Contemporary 3D digitization systems employed by reverse engineering (RE) feature ever-growing scanning speeds with the ability to generate large quantity of points in a unit of time. Although advantageous for the quality and efficiency of RE modelling, the huge number of point datas can turn into a serious practical problem, later on, when the CAD model is generated. In addition, 3D digitization processes are very often plagued by measuring errors, which can be attributed to the very nature of measuring systems, various characteristics of the digitized objects and subjective errors by the operator, which also contribute to problems in the CAD model generation process. This paper presents an integral system for the pre-processing of point data, *i.e*., filtering, smoothing and reduction, based on a cross-sectional RE approach. In the course of the proposed system development, major emphasis was placed on the module for point data reduction, which was designed according to a novel approach with integrated deviation analysis and fuzzy logic reasoning. The developed system was verified through its application on three case studies, on point data from objects of versatile geometries obtained by contact and laser 3D digitization systems. The obtained results demonstrate the effectiveness of the system.

## 1. Introduction

Current demands of a globalized market for constant shortening of development time for novel and increasingly complex products—in order to maintain a competitive edge—have made Reverse Engineering (RE) an extremely popular technique, to the point of being practically indispensable in some design problems. The market today dictates sudden and frequent re-design of products with emphasis on aesthetic and ergonomic features, which, in turn, require the ever more complex organic forms and shapes. These are most often very difficult—sometimes even impossible—to model using conventional CAD tools, which is why they first have to be modelled by sculpting (using clay, plaster, wood, *etc*.), and subsequently translated into CAD models using RE [1–5]. Besides, application of RE can substantially speed up and improve the process of re-design, regardless of whether it be own- or competition products. Also in the case of copying, or reproducing parts and products which are not accompanied by adequate engineering documentation there is practically no alternative to the RE technique [6–8].

Modern 3D digitization systems, which are employed by RE, feature ever-growing scanning speeds with the ability to generate large quantities of points in a unit of time [9–11]. Generally speaking, that is advantageous for the quality and efficiency of RE modelling. However, the huge number of point data, generated in the course of 3D digitization, can become a serious practical problem, later on, when the CAD model is generated. In addition, the 3D digitization process is very often plagued by measuring errors, which can be attributed to the very nature of measuring systems, various characteristics of the digitized objects and subjective errors by the operator, which also contribute to problems in the CAD model generation process [12–15].

Fundamental problems—caused by erroneous point data and a huge number of point data as the result of 3D digitization—are: deviations in shape of the resulting CAD model as compared to the original physical object, and impeded work with software applications for CAD model generation [2,16,17]. The process of surface model generation can be significantly slowed down, and in some extreme cases even brought to a complete halt, despite the high processing power of modern computers. Considering all this, one can conclude that the data pre-processing stage, which includes error filtering, smoothing and reduction of point data, is very important and almost unavoidable in every RE-system. In support of this claim, there is a fact that data pre-processing has for a number of years now been at the forefront of research, with the reduction of point data being one of central topics. The result is a number of developed systems for pre-processing of 3D digitization data, which are based on various approaches, and depend on RE methodology applied, that is, the 3D digitizing system for which they were developed [18–26].

In order to improve the quality and efficiency, artificial intelligence methods—fuzzy logic (FL), artificial neural networks (ANN) and genetic algorithms (GA)—have so far been implemented at various stages of RE, whereby they have been most frequently used for data pre-processing, or precisely, point data reduction. Philippe *et al*. [27] proposed an approach to predetermine the topology of a surface from the point cloud on the bases of FL. This method has been applied for the predefinition of the different main regions of a surface, in order to be able to divide the point cloud in different separate clouds corresponding to these regions. The main advantage of this method is that, for a given accuracy, the complexity of the surface is lower than a traditional method, and the computations are quite the same than those obtained when using traditional surface modelling algorithms. Chang and Lin [28] presented a system and methodology for on-line free form surface measurement via a scanning contact probe installed on a CNC machine. To improve the measuring stability and continuity, FL control, in lieu of traditional PID control, is employed. The presented measuring system’s main feature is that the number of measured points can vary with the change of surface curvature. Furthermore, Wu *et al*. [29] reported on fuzzy-clustering-based reduction method, where as an effective extension to the existing pure geometric reduction methods, a hybrid heuristic is introduced. It includes descriptions of samples’ fuzzy imperative attributes and fuzzy geometric attributes. Reduced points are favoured to gather in regions of high curvature and surface boundaries. Detailed features, which are particularly valuable for machining, can be well preserved. Peng and Loftus [30] developed an RE system which processes photographs using ANN. The network was trained on a representative training set in order to be able to recognize novel forms. Jun *et al*. [31] proposed a system which uses ANN for feature recognition in prismatic parts. Korosec *et al*. [32] presented an ANN approach used to find an optimal design space in order to improve precision and accuracy of RE models. Chen and Wang [33] reported on optimized re-triangulation based on GA. They described a method to generate optimized STL file (stereolithography CAD software file format developed by *3D Systems*) with reduced point data generated by laser scanning. The optimization is performed by reducing the number of triangles in STL file using triangles’ vector normals. After extraction of triangles, re-triangulation is performed as in other reduction methods. Percoco and Spina [34] analysed the application of GA in reduction of point data in polygonal models. Another example of application of GA in RE is given by Xiaodong *et al*. [35]. They presented an approach to simplification of meshed models using GA. Moreover, Galantucci *et al*. [36] applied a hybrid approach, which combines ANN and GA, for the automatic registration of point clouds with free-form shapes.

Amidst a number of developed approaches to point data reduction, three of them stand out as dominant: reduction by sampling, polygonal reduction, and mesh reduction [18,19,21,37]. Point data reduction with the “*cross-sectional*” RE methodology, is primarily based on the so called sampling methods, amongst which the most popular are: factor-, spatial-, chord-, angle- and height methods [18,19,21,38,39]. All these methods use different parameters as criteria for point data reduction. However, the key indicator of quality of point data reduction methods is the ratio between the resulting deviation (of the cross-sectional curves, *i.e*., surface model) and the level (percentage) of data reduction. Accordingly, the underlying question of the so far investigations in this research area can be summarized as: *How to increase the level of data reduction*, *while simultaneously decreasing the error*, i.e., *the level of deviation?*

The basic research objective of the study is the development of a system for quality pre-processing of 3D digitization data, which would satisfy current standards for accuracy in mechanical engineering. Special emphasis is placed on the development of a sub-system for the reduction of point data. The research is primarily focused on cross-sectional RE methodology and the results of digitization generated by the two, presently most widely used, systems for 3D digitization in mechanical engineering—contact scanning and optical triangulation. Bearing in mind that, from user’s perspective, the abstract nature of parameters is a significant problem in reduction of point data using sampling methods, the second objective of this study is to develop an approach which would allow more user-friendly and intuitive interaction. The result is the developed integral system for pre-processing of point data based on cross-sectional RE approach presented in this paper. The system, provisionally named **Sy**stem for **Pre**-processing by **F**uzzy logic (SyPreF), has a modular structure with a total of five modules, where major emphasis was placed on the module for point data reduction designed according to a novel approach. Building on the weak spots and deficiencies of current approaches to reduction of point data by sampling methods—*i.e.*, the lack of information on the level of deviation in reduced point clouds and necessity to employ parameters which are abstract to user—a novel approach was developed for analysis of the level of deviation of the reduced point cloud in comparison with the initial point cloud. Beside the additional improvement of the deviation/mean level of reduction ratio, implementation fuzzy logic allowed a more user-friendly and intuitive application. The second part of the paper presents SyPreF’s verification results obtained through its application in three case studies, defined on the basis of geometry complexity, dimensions and applied system for 3D digitization.

## 2. Development of the System for Point-Data Pre-Processing

**Figure 1.**
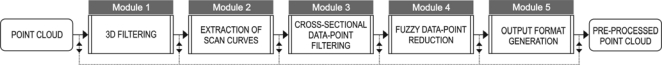
General model of the SyPreF system.

The system developed for point data pre-processing, which is primarily intended for the needs of reverse engineering modelling on the basis of the “cross-sectional” methodology, is designed on the modular principle and consists of (Figure 1):

1. Module for 3D filtering of points (errors);
2. Module for extraction of point data in cross-sections;
3. Module for filtering and smoothing of point data in cross-sections;
4. Module for reduction of point-data based on fuzzy logic;
5. Module for formatting of output data.

### 2.1. Module 1—3D Filtering

3D digitization most often results in numerous unwanted points. These points frequently belong to objects which surround the object being digitized, such as fixtures, measurement table, or some other part of the assembly to which the digitized part belongs. However, in the case of non-contact methods, such as the laser triangulation, those points can originate from objects located further away. To some extent, the unwanted points can also be the result of measurement errors (due to operator errors, system-specific errors and/or errors due to specific nature of the digitized object, some external disturbance *i.e*., vibrations), *etc*. Those points, as described in the Introduction section, have to be eliminated in order to maintain quality of surface reconstruction. The module for 3D filtering of errors was developed to allow elimination of unwanted points. The module consists of three tools, which perform:

- Volumetric filtering;
- Filtering by segmented line;
- Elimination of particular points (by selection).

**Figure 2.**
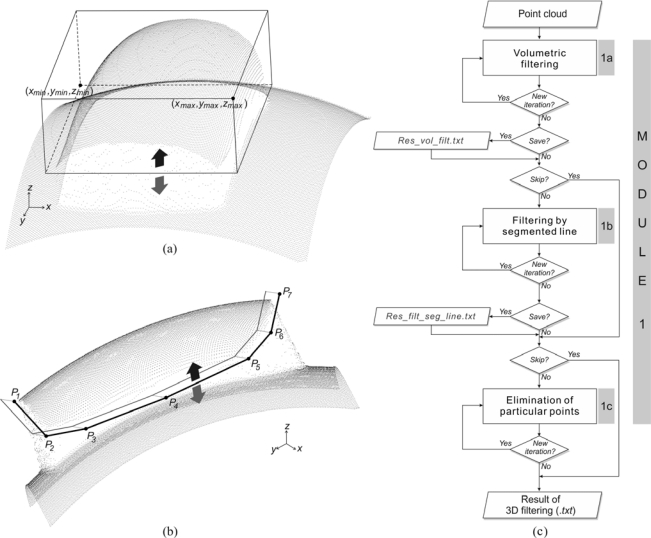
(**a**) Principle of the volumetric filter. (**b**) Principle of the segmented line filter. (**c**) Algorithm of the module for 3D point (error) filtering.

The volumetric filtering method is based on forming a rectangular volume (Figure 2(a)) defined by the length, width and height of scanning (*x-, y*-, and *z*-axis). Any point data falling outside/inside (optional user choice) this volume are considered as unwanted and eliminated. This is a rough method and is used to eliminate extreme errors or point data belonging to fixtures and the measuring table, which may occur during 3D digitization.

The segmented line filter is based on staggered lines which are composed of line segments, defined by the knot points (*P_i_*) in the selected plane *x-y*, *x-z* or *y-z.* After the filter line has been generated, point data are eliminated from one of its sides on the bases of the user choice (Figure 2(b)).

The third tool allows individual selection of a larger number of points which could not be eliminated using the first two tools. Selection is performed in a graphical mode, from the point cloud. The three tools can be used each by itself, or in any other combination, iteratively, as shown by the algorithm in Figure 2(c).

### 2.2. Module 2—Extraction of Point Data in Cross-Sections

**Figure 3.**
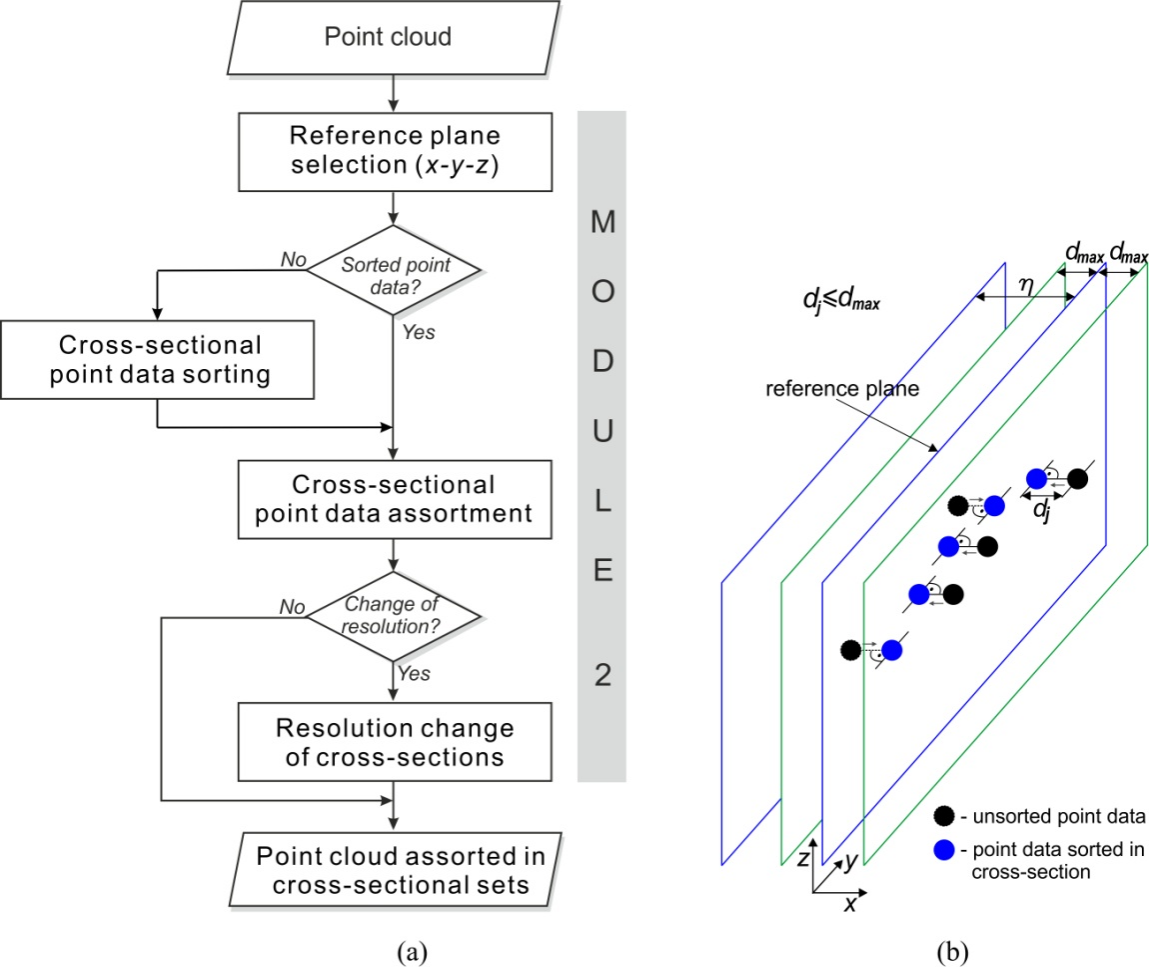
(**a**) Algorithm of module for extraction of cross-sectional curves. (**b**) Principle of the integrated approach for cross-sectional point data extraction from unsorted point clouds.

Bearing in mind that SyPreF is dedicated to RE modelling based on cross-sectional methodology, prior to pre-processing (filtering, smoothing, and reduction) it is necessary to select and sort the points from the 3D point cloud—by cross-sections. Accordingly, within this module (points belonging to particular 2D cross-sectional curves are selected into a separate set Figure 3(a)), and then sorted within each of the scanned curves. The selection can be performed in all three directions—*i.e*., along *x*, *y*, and *z* axes.

However, if the input point cloud is unsorted—*i.e.*, point data is not sorted into sections or scanned lines—the system provides a sorting option. Point data is sorted within cross-sections parallel to the selected reference plane, by projecting all the points that pertain to defined tolerance zone. The number of cross-sections is directly related to the defined resolution (*η*), while the tolerance zone represents the band whose width is defined by the maximum allowed shortest distance (*d_max_*) of the points from both sides of the reference planes (Figure 3(b)). It is obvious that a wider tolerance zone implies lower accuracy of the sorting.

This module also provides an option to change the resolution of the cross-section, *i.e*., the scanned lines. This option can be very useful in case of very densely scanned lines, when the geometric complexity of the scanned object does not require large cross-sectional resolution.

### 2.3. Module 3—Cross-Sectional Filtering/Smoothing of Point Data

The function of this module is two-fold: filtering and smoothing of point data in cross-sections (scanned lines). It is dedicated to elimination of errors left after 3D filtering, as well as elimination of noise which affects the quality of the resulting surface model. The module is based on four tools:

- Elimination of points at the ends of scanned curves;
- Filtering by the angle method;
- Filtering/smoothing by the method of median;
- Smoothing by the method of mean.

The first tool (a) is dedicated to elimination of points at the ends of scanned curves. These points are often problematic because they are the result of unwanted contacts between the sensor and the fixture, measurement table and similar objects, but were not eliminated in the process of 3D filtering. User is called to define the number of points that should be eliminated at the ends.

**Figure 4.**
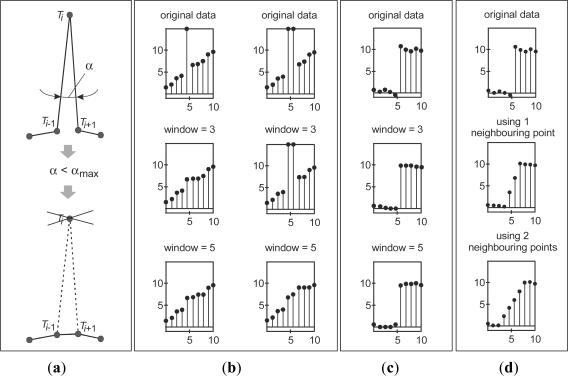
Principles and effects of methods for cross-sectional filtering/smoothing. (**a**) Method of angle. (**b**) Elimination of impulse noise by method of median. (**c**) Point data smoothing by median filter in cases of stepped increments. (**d**) Point data smoothing by mean filter in cases of stepped increments.

The angle method (b) is dedicated to elimination of outliers, *i.e.*, impulse noise, and the principle is presented in Figure 4(a). The pre-defined angle value in SyPreF is *α* = 10°, and it can be optionally changed according to user’s needs.

The principle of the method of median is mathematically defined as:

(1) y(i)=Med[x(i−N), x(i+N)]

where *x*(*i*) is input data, *y*(*i*) output data, and *N* is a half width of the filter window (the number of points which is considered on one side of the analyzed point) [40].

The principle of the method of mean is based on statistical mean of the specified data array *x*(*i* − *N*, ..., *i* + *N*) defined, as in median filter, by the filter window width (*N*):

(2) y(i)=Mean[x(i−N), x(i+N)]

The method of median can also be used for elimination of outliers as well as for point data smoothing, while the method of mean is exclusively used for smoothing [21]. As presented in Figure 4(b), the median filter can be useful in elimination of impulse noise. In contrast to the angular filter, this filter retains the analyzed point in the array, but it changes the y-coordinate. Therefore, its use may make more sense in cases of lower density point data from geometrically more complex objects.

Moreover, the median filter tends to preserve the shape even in cases of stepped increments (Figure 4(c)). This is its main advantage compared to the mean filter (Figure 4(d)), which is very rarely used, and mainly in cases of noisy point data for shape refinement. However, as the effects of this two filters’ application are strongly dependant on filter window’s width (*N*), it must be considered very seriously in order to avoid unwanted filtering actions, above all the shape modification. It is important to note that larger value of *N* with median filter, in general, decreases the possibility of the mentioned unwanted filtering actions.

**Figure 5.**
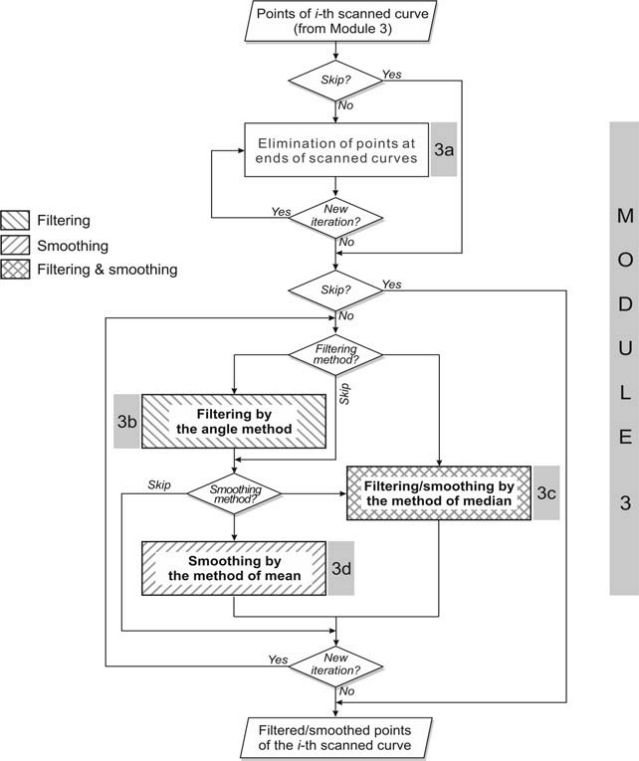
Algorithm of module for cross-sectional filtering/smoothing.

Figure 5 presents the algorithm of the module’s software implementation. Each tool is applied on user’s request with the option of accepting/changing pre-defined parameter values. The process is infinitely iterative and reversible, *i.e.*,—results of tools’ application that are graphically presented, can be cancelled by the user.

### 2.4. Module 4—Fuzzy-Based Reduction of Point Data

**Figure 6.**
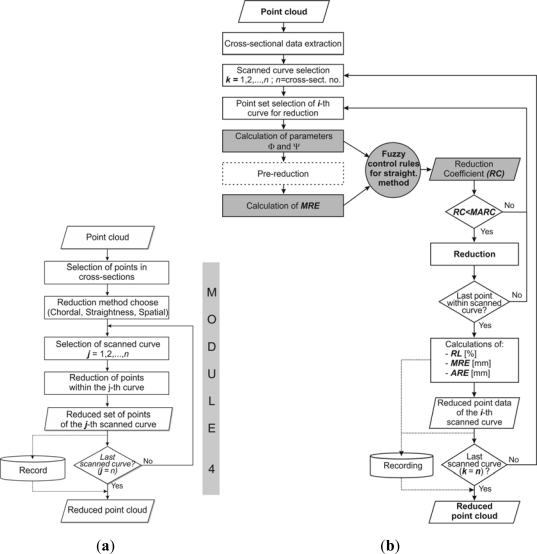
(**a**) Algorithm of the developed module for point data reduction based on fuzzy logic. (**b**) Algorithm of the fuzzy-logic-based point data reduction by method of straightness.

This module (Figure 6(a)) represents the most sophisticated segment of the presented system. The developed approach is based on improvement of the existing decision-making procedures by application of fuzzy logic and introduction of an additional parameter—*maximum allowed deviation*. The approach is integrated into three sampling-based methods for point data reduction—the chordal method, straightness method, and spatial method [19,21,40].

To eliminate the problems which stem from the specific values of decision-critical input parameters entered by the user, and create a more user-friendly system, a new, synthetic parameter was introduced under the name *reduction coefficient* (*RC)*, and its maximum allowed value was defined as *maximum allowed reduction coefficient* (*MARC)*. For all three methods the RC parameter was derived based on method-specific parameters (which is given a detailed explanation within method descriptions to follow), and an additional input parameter *maximum reduction error (MRE)*, *i.e*., the maximum allowed reduction error (*MARE)*, introduced to allow the maximum reduction error to be controlled.

Detailed descriptions of software procedures for the method of straightness is given below, while for other two integrated methods—spatial and chordal—they can be found in [41] and [42], respectively.

**Figure 7.**
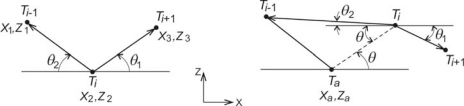
Principle of point data reduction based on straightness [40].

The straightness method for reduction of point data (the mathematical background of the method is explained by relations (3) and (4) and illustrated in Figure 7) is based on two parameters *Φ* and *Ψ* [40]. These parameters, together with parameter *MRE*, were used as input variables for the fuzzy logic system used for decision-making. The only difference being that *Φ* and *Ψ* were used not as two independent parameters, but rather as a sum of values. Such approach was allowed by the fact that both parameters were compared against the same reference value—*the defined level of straightness* (*DLS*). This means that by simply defining the boundary values for fuzzy system input, *Φ* ≤ *DLS* and *Ψ* ≤ *DLS*, the fuzzy rules can be fed the sum *Φ* + *Ψ* as a single parameter. For sake of simplicity, this parameter is further on denoted as *Ω*.

(3) $$\Phi =\left|\theta_{1}+\theta_{2}\right|=\left|{\text{tan}}^{-1}\;\left(\frac{z_{3}-z_{2}}{\left|x_{3}-x_{2}\right|}\right)+{\text{tan}}^{-1}\;\left(\frac{z_{1}-z_{2}}{\left|x_{1}-x_{2}\right|}\right)\right|$$

(4) $$\Psi =\left|\theta +\theta_{1}\right|=\left|{\text{tan}}^{-1}\;\left(\frac{z_{1}-z_{a}}{\left|x_{1}-x_{a}\right|}\right)+{\text{tan}}^{-1}\;\left(\frac{z_{3}-z_{2}}{\left|x_{3}-x_{2}\right|}\right)\right|$$

The procedure for fuzzy logic-based reduction is illustrated in Figure 6(b) and comprises the following: *MARE* is defined to allow the software application to compute *MARC*, set of points is selected for computation of parameters (an array of four consecutive points) and parameters *Φ* and *Ψ* are computed, *i.e*., their sum *Ω =Φ* + *Ψ*. Preliminary elimination of the second point in sequence is performed in order to calculate the *MRE*. Based on the computed parameters, and the fuzzy rules defined, *RC* is computed. By comparing the *RC* against the predefined *MARC*, a decision is made whether to eliminate or to retain the analyzed point. In case that the second point in the array is eliminated, the new set for analysis is composed of points 1, 3 and 4 from the previous set, and the next point in the scanned array, while in the case when no elimination occurred, the new set is composed of points 2, 3, and 4 from the previous set, and the next point in the scanned array. After the last point in the scanned array has been selected, and the first run of the reduction procedure is finished, a change of direction in the selected point set is performed which means that the last point in the scanned array of the last run, becomes the first point in the next run, which makes the process of reduction more even. The process is re-iterated on a reduced scanned point array until it is possible to eliminate at least one point in a single run.

**Figure 8.**
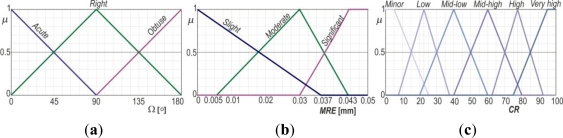
(**a**) Membership function of input state variable *Ω*. (**b**) Membership function of input state variable *MRE*. (**c**) Membership function of output state variable *RC*.

The input consists of two state variables—*Ω* and *MRE* (Figure 6(b)) whose real values are fuzzified *into fuzzy sets* with appropriate input spaces, where the fuzzy sets with input values are defined by appropriate membership functions. Triangular membership function was used for all membership functions. Considering the previously mentioned character of *Φ* and *Ψ,* as well as the practical experience with their values, for the input variable *Ω* an input space of 0° to 2·90° was defined, that is, from 0° to 180°. This space was segmented *into three fuzzy sets* using linguistic terms to describe angle characters: *sharp*, *right* and obtuse(Figure 8(a)).

Input space for the state variable *MRE* is defined on the basis of real-application experience with *MARE* = 0.05 mm, as the pivotal parameter and was segmented into three fuzzy sets—*slight, moderate and significant* (Figure 8(b)). The output from this fuzzy system is variable *RC* (a non-dimensional value) which, for simplicity sake, has been allotted an output space from 0 to 100. To allow finer control of *RC* parameter, the input space was segmented with finer resolution, resulting in a total of nine fuzzy sets denoted with linguistic qualifiers—*minor, very low, low, medium-low, medium, medium-high, high, very high*, and *huge* (Figure 8(c)). The knowledge base comprises a total of nine control fuzzy rules:

1. If (Ω is *Acute*) and (MRE is *Slight*) then (RC is *Huge*)
2. If (Ω is *Right*) and (MRE is *Slight*) then (RC is *Very-high*)
3. If (Ω is *Obtuse*) and (MRE is *Slight*) then (RC is *High*)
4. If (Ω is *Acute*) and (MRE is *Moderate*) then (RC is *Mid-high*)
5. If (Ω is *Right*) and (MRE is *Moderate*) then (RC is *Mid*)
6. If (Ω is *Obtuse*) and (MRE is *Moderate*) then (RC is *Mid-low*)
7. If (Ω is *Acute*) and (MRE is *Significant*) then (RC is *Low*)
8. If (Ω is *Right*) and (MRE is *Significant*) then (RC is *Very-low*)
9. If (Ω is *Obtuse*) and (MRE is *Significant*) then (RC is *Minor*)

### 2.5. Module 5—Output Data Formatting

The role of this module is to adjust and prepare the results of pre-processing by formatting them into form acceptable to some of the software systems for surface reconstruction. The proposed model supports two point data formats: PTS and IBL.

### 2.6. Implementation Details

**Figure 9.**
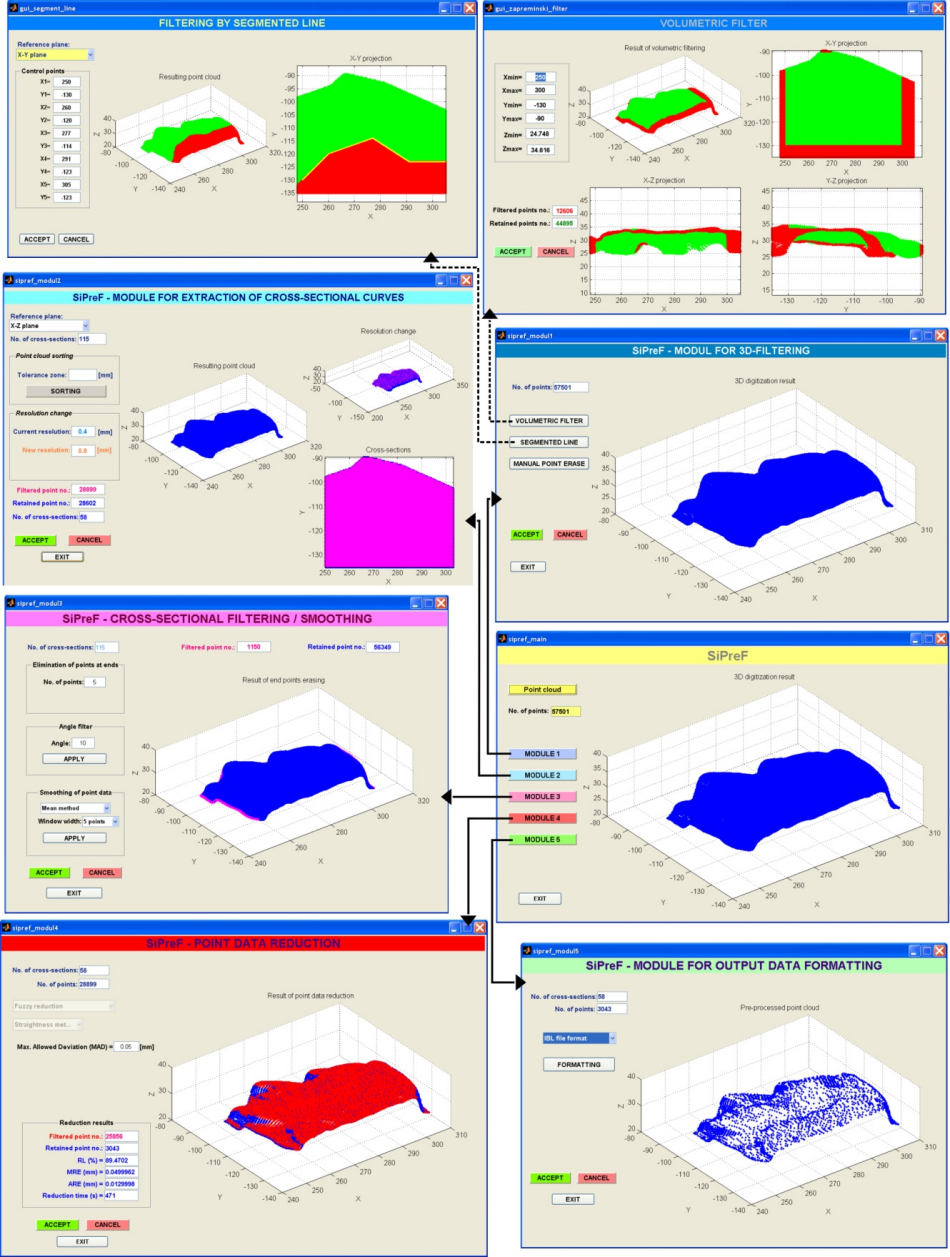
SyPreF module’s interfaces.

In realization of the SyPreF point data pre-processor, conventional programming tools as well as artificial intelligence (fuzzy logic) tools were used. SyPreF was developed in *Matlab (MathWorks) 7.2 (Release 2006a)*, based on prerequisites explained in the previous sections. Graphical user interfaces of the modules are presented in Figure 9.

## 3. Verification Results

The system for pre-processing of point data, described in previous chapter, was verified on three characteristic case studies:

1. timing belt tooth,
2. human face model and
3. sports glasses lens.

In the first case study 3D digitization was performed by laser scanning, while in other two case studies it was accomplished by contact scanning.

### 3.1. Case Study 1

**Figure 10.**
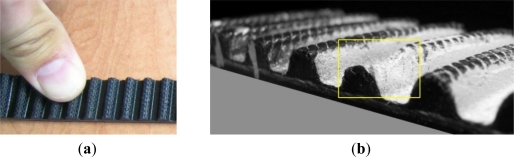
(**a**) Segment of the timing belt. (**b**) Framed part of the tooth with a factory error.

**Figure 11.**
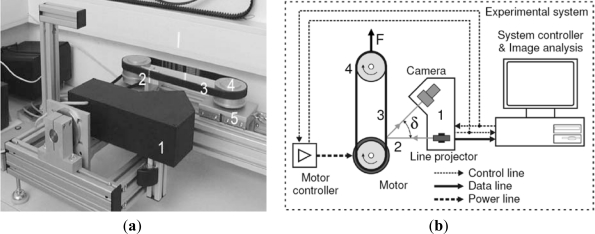
Non-contact 3D digitization system (1-laser profilometer module, 2-laser plane, 3-belt, 4-pulleys, 5-movable cart). (**a**) Photo of the system. (**b**) Schematic diagram of the system.

**Figure 12.**
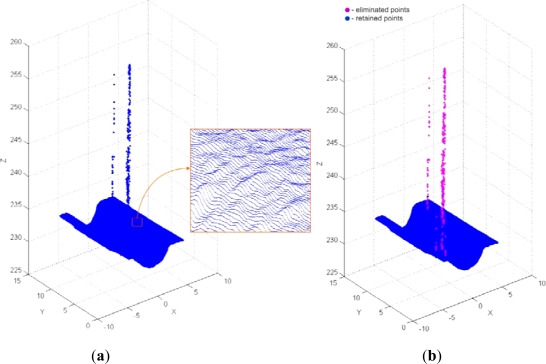
(**a**) Result of 3D digitization (with magnified detail). (**b**) Result of filtering by the method of angle.

The first case study which was used to test the SyPreF included a timing belt tooth (Figure 10(a)), specifically—a tooth part which has a factory error (Figure 10(b)). Specific features of this case study are very small object dimensions and non-contact 3D digitization, which was performed by a laser triangulation sensor (Figure 11). The resulting point cloud (Figure 12(a)) consists of 1,482,000 points and contains substantial errors which are inherent to this non-contact method, and it also features high resolution (5 μm).

**Figure 13.**
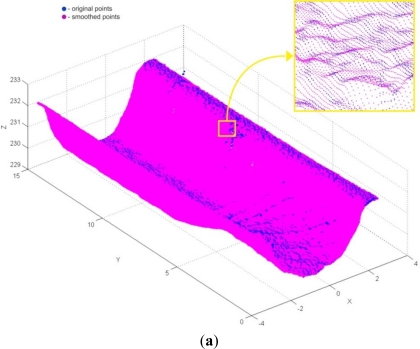

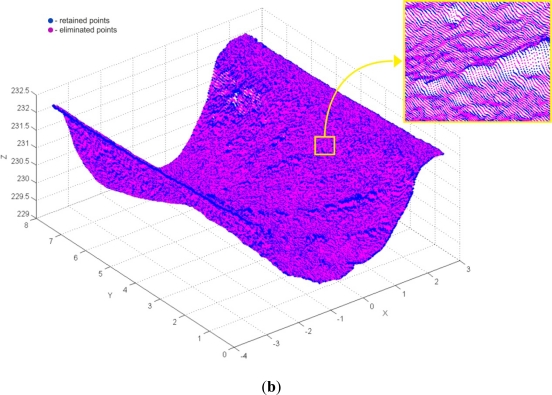
(**a**) The result of point data smoothing. (**b**) The result of point data reduction.

Point-data pre-processing was started by the application of the tools for cross-sectional filtering/smoothing of point data. Primarily filtering by the angle method (*α* = 10°) was conducted. The result was 489 filtered and 1,481,511 retained points (Figure 12(b)). Bearing in mind the application of the powder layer sprayed on the object before the 3D digitization, smoothing of point data by the median method (with *N* = 5) was performed after the filtering (Figure 13(a)).

The next phase of pre-processing was the extraction of point data in cross sections. In order to efficiently reconstruct the surface model, the cross-sectional resolution was changed from 5 to 10 μm. In this way the number of cross-sections was lowered from 3,000 to 1,500, which in turn decreased the number of points to 494,332.

**Figure 14.**
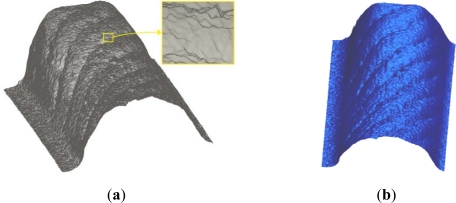
(**a**) Generated cross-sectional curves. (**b**) Created surface model.

A special focus here was on the tooth part with a factory error, so the frontal segment of the point data was extracted from the whole by application of a volume filter. According to this, the point data reduction was performed on the point cloud consisting of 263,791 points in 801 cross-sections. The reduction was performed by the fuzzy-chordal method with *MAD* = 0.01 mm. Results are shown in Table 1 and in Figure 13(b). The generated cross-sectional curves and the resulting surface models are shown in Figure 14.

### 3.2. Case Study 2

**Figure 15.**
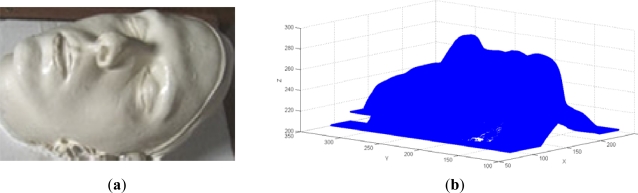
(**a**) Human face model. (**b**) Result of 3D digitization.

The second verification case study was performed on a human face cast model (Figure 15(a)). Digitization was performed by contact scanning using a *Cyclon II—Renishaw* system, and contains 905,931 points (Figure 15(b)). Bearing in mind moderate complexity of the point data from the filtering aspect, this case study was used firstly to verify the module for reduction of point data.

**Figure 16.**
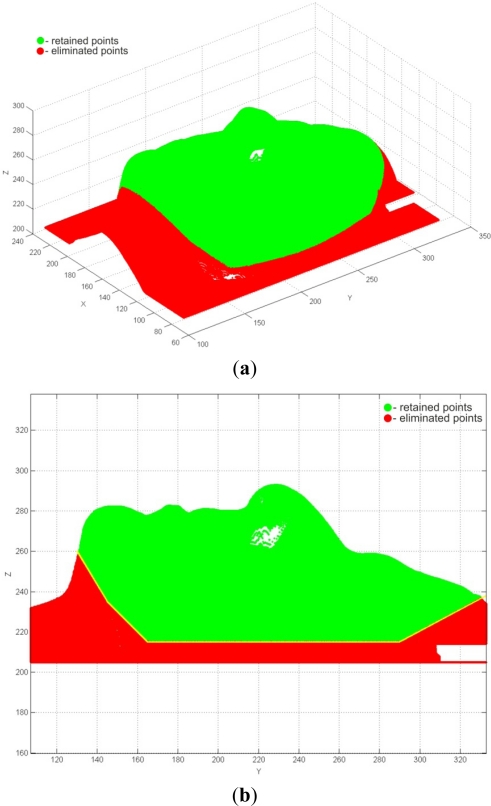
Results of filtering by segmented line. (**a**) 3D view. (**b**) *y*-*z* plane view.

Point-data pre-processing was started by the application of the tools for 3D filtering of point data. Considering that the model is symmetric about the *x*-axis (*i.e*., *y*-*z* plane) point-data pre-processing was started by applying 3D filtering with a segmented line filter and a total of four segments. Coordinates of the knot points defining the filter line’s segments are given in Table 2. This resulted in a 351,292 filtered points (Figure 16).

**Figure 17.**
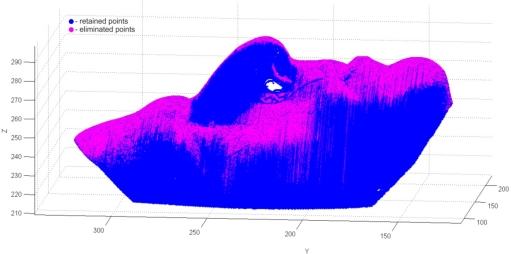
The result of point data reduction.

The next phase of point data pre-processing was the reduction. The point cloud prepared for reduction contained 564,612 points in 979 cross-sections. The reduction was performed by the fuzzy-straightness method with *MAD* = 0.04 mm. Results are given in Table 3 and in Figure 17.

**Figure 18.**
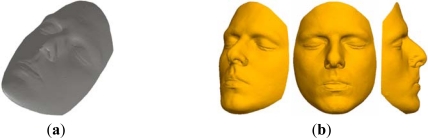
(**a**) Generated cross-sectional curves. (**b**) Generated surface model.

The surface model was generated based on the reduced point data in IBL format. Generated cross-sectional curves and resulting surface models are shown in Figure 18. Certain problems noticeable at the base of the nose can be attributed to a very small number of points in that area produced by 3D digitization.

### 3.3. Case Study 3

**Figure 19.**
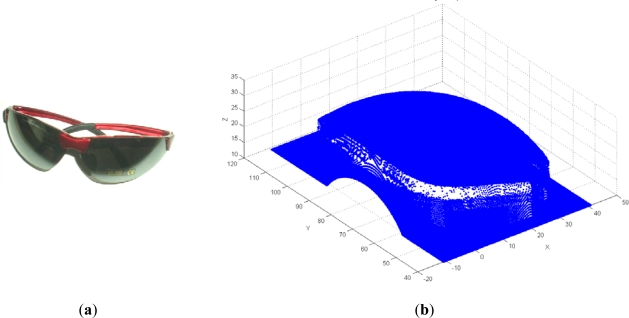
(**a**) Sports glasses lens. (**b**) The results of 3D digitization.

The third case study involved a sports glasses lens (Figure 19(a)). The choice of this part, which due to ergonomic intent is of a relatively simple geometry, was motivated by the complexity of the digitized data (Figure 19(b)) which requires adequate fixture and locating. 3D digitization was performed by a contact system *Cyclon II—Renishaw*, resulting in a total of 412,111 points, of which a large number represent error-points which actually belong to the fixture and measuring table.

**Figure 20.**
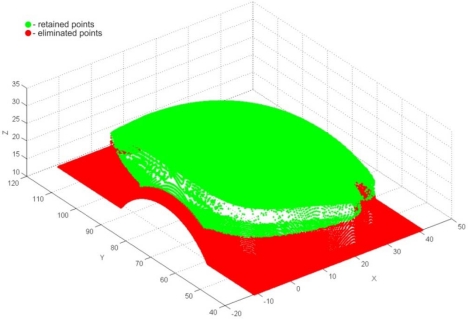
The result of volumetric filtering.

**Figure 21.**
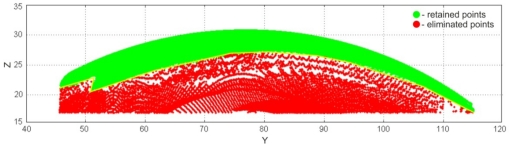
The result of filtering by segmented line.

**Figure 22.**
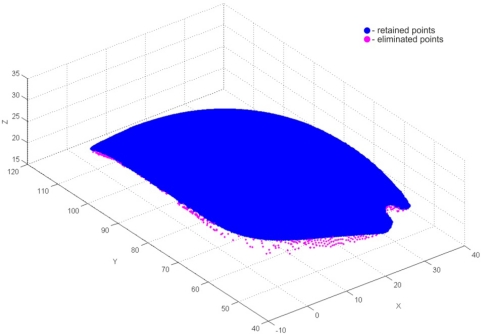
The result of manual elimination of points.

The very first pre-processing phase was the 3D filtering of resulting point data, which included volumetric filtering, filtering by segmented line, and elimination of individual points. Parameters used for volumetric filtering are listed in Table 4. The result was 175,315 filtered points, and 236,796 remaining points (Figure 20). Filtering by segmented line was performed in *y-z* plane. Knot points defining the segmented line are listed in Table 5 and the obtained result (filtered 12,013 and 224,783 remained points) is shown on Figure 21. In this case, the geometric form and spatial orientation of object were the cause of a larger number of left-over point-errors—a total of 367 points were eliminated manually, thus leaving 224,416 points (Figure 22).

**Figure 23.**
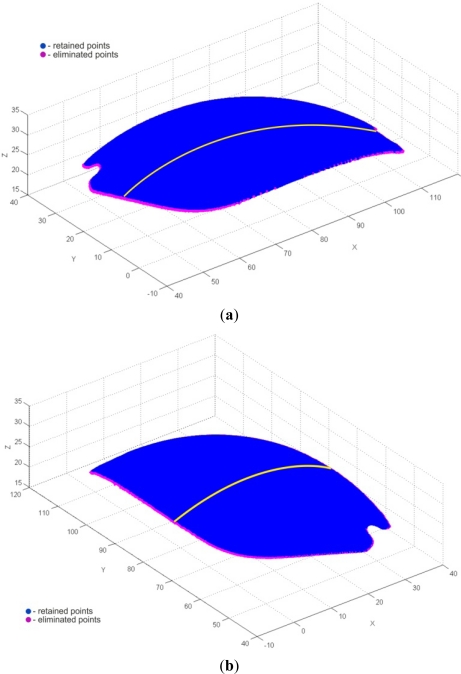
Elimination of end-points in cross-sections. (**a**) 1st iteration—along *y* axis. (**b**) 2nd iteration—along *x* axis.

Cross-sectional filtering/smoothing of point data was performed in the next phase of pre-processing and comprised elimination of end points in two iterations—1st along the *y* axis, and 2nd along the *x* axis (due to the disposition of points it was possible to eliminate end-points along two axes). This tool was applied because of a higher probability of error-points around the object’s rims, caused by the transition of the digitization sensor probe from object to fixture and measuring table. Within the 1st iteration, along *y* axis, a total of 2,586 points (3 points at each end in all of the 429 cross-sections) were eliminated (Figure 23(a)). Likewise, in the 2nd iteration along *x* axis, a total of 2,748 points (2 points at each end in all of the 687 cross-sections) were eliminated, so the resulting point cloud contained 219,082 points (Figure 23(b)).

Bearing in mind a relatively simple object geometry, in the next phase of pre-processing cross-sectional resolution was changed from 0.1 to 0.2 mm along *y* axis (*x*-*z* plane was chosen as the reference plane). In this way, the number of cross-sections was lowered from 429 to 214, which lowered the total number of points from 219,082 to 109,528.

**Figure 24.**
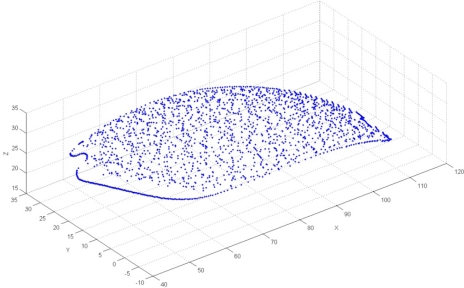
The result of point data reduction.

In the final phase of pre-processing, reduction of point data was performed. Fuzzy-chordal reduction method was chosen, with *MAD* = 0.03 mm. The results of reduction are presented in Table 6, and in Figure 24.

**Figure 25.**
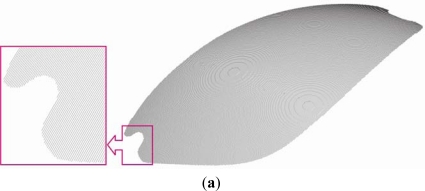

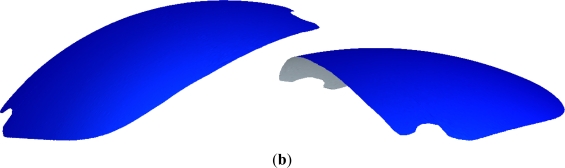
(**a**) Generated cross-sectional curves. (**b**) Surface model.

As in previous two case studies, a surface model was generated by automated generation of cross-sectional curves from the prepared point clouds in IBL format. The generated cross-sectional curves and the resulting surface model are shown in Figure 25.

## 4. Discussion

This part deals with the analysis of the obtained results in terms of point data filtering and point data reduction. Processing time is discussed in particular with reduction, since it is negligible in point data filtering.

In terms of filtering, the first and third case studies were more demanding, which imposed the application of a combination of tools for point data filtering. In the first case study, which is characterized by a large number of impulse errors typical for a laser triangulation digitization, filtering process was carried out without the need for manual user’s work, primarily due to efficient outliers’ filtering by the method of angle. The third case study is characterized by the need for using multiple filtering tools. Although the efficiency of the volumetric filter, segmented line filter and the end point filter showed satisfactory, object’s geometry in this case imposed a need for manual filtering of individual points by the user. Unlike in previous cases, in the second case study is—due to object’s geometry and point cloud’s features—filtering was effectively carried out using only the segmented line filter.

Related to point data reduction, it has to be emphasized that the presented approach allows the user to conduct the reduction process qualitatively by defining the desired value of the maximum tolerance, *i.e.*, maximum allowed deviation. This is—compared to the quantitative approaches based on the reduction percentage definition or approaches based on setting (usually abstract) parameters’ values—more intuitive in terms of the expected quality of the reconstructed model. The developed approach is validated through its implementation in the three different sampling methods for point data reduction, showing—as demonstrated through three case studies—analogous efficiency in all three methods. Common features of the first and the second case studies is the objects’ geometry, characterized by significant difference in *z*-coordinate values within cross-sectional point data arrays, which implied an intermediate reduction levels. However, in the first case study for a four times lower level of *MAD* (0.01 *versus* 0.04 mm) achieved level of reduction was higher for about 12%. Nevertheless, it should be considered that the overall dimension of the object in the first case study was 25 times smaller, as well as that the object’s surface from the second case study was smoother. The object of a third case study is characterized by the simpler geometry that implicated a very high level of reduction of almost 98%. This information should be considered with the facts that this object was approximately three times smaller from the object in the second case study, as well as that reduction was performed with lower level of *MAD* for 0.1 mm (0.03 *versus* 0.04 mm). Smaller size of the object implies a finer resolution, *i.e.*, higher density of point data, which generally provides a higher reduction level for a given level of MAD. As for the geometric complexity of the scanned object, a smoother object’s surface implies the achievement of higher reduction level.

The processing time can be taken as a conditional drawback of the presented reduction approach. Because of the high dependence of the reduction process on: object’s geometry, level of *MAD*, and (*cross-sections no.*)/(*no. of points in cross-section*) ratio, it is not possible to express the average speed reduction, but it can be roughly estimated at about 350,000 points per hour, for *MAD* of about 0.05 mm. The processing time in the presented case studies ranged from about 25 to 95 min, noting that the results were achieved on a PC configuration of medium-high performance. With respect to the mathematical background of the developed algorithms, one can expect better results on more powerful PC platforms.

The attribute “conditional”, at a stated time demanding as a drawback in previous paragraph, is specified taking into account the benefits in the process of creating surface models based on reduced point clouds. Direct benefits include a significant speed increase of the surface model’s generation process as well as enormously increased stability of the process (referring to software crash-down). The former imply time savings which are usually higher than needed to compensate time spent on the reduction of point clouds. Moreover, much greater working flexibility (e.g., in solidification, *etc*.) with models generated from the reduced point data (which stems from their less memory size) should be mentioned as an indirect benefit.

## 5. Conclusions

Presented in this paper is an integral system for pre-processing of point data based on a cross-sectional RE approach. The system, provisionally entitled SyPreF, is of modular structure with a total of five modules containing the developed software tools for: 3D filtering, extraction of point data in cross-sections, filtering and smoothing of point data within cross-sections, reduction of point data and generation of output formats for application in dedicated software for surface reconstruction.

Major emphasis was placed on the module for point data reduction, which was designed according to a novel approach with integrated deviation analysis and fuzzy logic reasoning. Building on the weak spots and deficiencies of current approaches to reduction of point data by sampling methods—*i.e*., the lack of information on the level of deviation in reduced point clouds and necessity to employ parameters which are abstract to user—a novel approach was developed for analysis of the level of deviation of the reduced point cloud in comparison with the initial point cloud.

The approach was tested with three different methods of sampling—chord, straightness and spatial—showing a significant improvement of the maximum deviation/level of reduction ratio, *i.e*., improvement of average deviation and level of reduction. Beside the improvement of the deviation/mean level of reduction ratio, the novel approach allowed a more user-friendly and intuitive application as the reduction process is controlled by simply entering the deviation tolerance, allowing the user to gain better insight into the quality of the reduced point cloud.

Verification of the proposed pre-processing system was conducted through its application in three case-studies selected on the basis of geometry complexity, dimensions and applied system for 3D digitization. Achieved results—high reduction levels and low deviations of reconstructed models—confirm the system’s effectiveness on point data from parts of versatile geometries, obtained by contact and laser triangulation digitization systems. Additionally, productivity and speed of surface reconstruction based on pre-processed point clouds are significantly increased, maintaining stability of process on an average hardware configuration.

Future research will be oriented towards further development through implementation of additional modules/tools (e.g., for point data segmentation and registration) as well as on further advancement of point data reduction through improvement of the calculation speed and development of an expert system for proposing an optimal method for the given point data.

## Figures and Tables

**Table 1.** The results of point data reduction.

**Parameter**	**Value**
MAD	0.01 mm
Maximum error	0.00968 mm
Average error	0.00159 mm
Reduction level	65.69 %
Number of retained points	90,494
Reduction time	73.38 min *

* Obtained on PC configuration: *Intel i7-870 (8M Cache, 2.93 GHz); DDR3 Kingston 8 GB 1,333 MHz.*

**Table 2.** Parameters of the applied segmented line filter.

**Knot point**	***y*-coordinate**	***z*-coordinate**
*P*_1_	130	260
*P*_2_	145	235
*P*_3_	165	215
*P*_4_	290	215
*P*_5_	333	238

**Table 3.** The point data reduction results.

**Parameter**	**Value**
MAD	0.04 mm
Maximum error	0.03993 mm
Average error	0.00491 mm
Reduction level	53.29 %
Number of retained points	275,811
Reduction time	95.45 min *

* Obtained on PC configuration: *Intel i7-870 (8M Cache, 2.93 GHz); DDR3 Kingston 8 GB 1,333 MHz.*

**Table 4.** Parameters used for volumetric filtering.

**Parameter**	**Value**
*x*_min_	−8.5
*x*_max_	36
*y*_min_	43
*y*_max_	116
*z*_min_	17
*z*_max_	35

**Table 5.** Parameters of the applied segmented line filter.

**Knot point**	***y*-coordinate**	***z*-coordinate**
*P*_1_	41.6	20.0
*P*_2_	51.2	23.1
*P*_3_	50.8	20.8
*P*_4_	61.1	23.9
*P*_5_	73.8	27.0
*P*_6_	80.2	27.2
*P*_7_	88.7	26.7
*P*_8_	94.0	25.3
*P*_9_	106.7	21.4
*P*_10_	116.8	16.2

**Table 6.** The results of point data reduction.

**Parameter**	**Value**
MAD	0.03 mm
Maximum error	0.02835 mm
Average error	0.00265 mm
Reduction level	97.82 %
Number of retained points	2,062
Reduction time	23.05 min *

* Obtained on PC configuration: *Intel i7-870 (8M Cache, 2.93 GHz); DDR3 Kingston 8 GB 1,333 MHz.*
